# Keap1, the cysteine-based mammalian intracellular sensor for electrophiles and oxidants^☆^

**DOI:** 10.1016/j.abb.2016.08.005

**Published:** 2017-03-01

**Authors:** Albena T. Dinkova-Kostova, Rumen V. Kostov, Peter Canning

**Affiliations:** a Division of Cancer Research, School of Medicine, University of Dundee, Scotland, UK; b Department Pharmacology and Molecular Sciences and Department of Medicine, Johns Hopkins University School of Medicine, Baltimore, MD, USA; c Weatherall Institute of Molecular Medicine, John Radcliffe Hospital, University of Oxford, Oxford, UK

**Keywords:** Keap1, Nrf2, Oxidants, Electrophiles, Cysteine sensor

## Abstract

The Kelch-like ECH associated protein 1 (Keap1) is a component of a Cullin3-based Cullin-RING E3 ubiquitin ligase (CRL) multisubunit protein complex. Within the CRL, homodimeric Keap1 functions as the Cullin3 adaptor, and importantly, it is also the critical component of the E3 ligase that performs the substrate recognition. The best-characterized substrate of Keap1 is transcription factor NF-E2 p45-related factor 2 (Nrf2), which orchestrates an elaborate transcriptional program in response to environmental challenges caused by oxidants, electrophiles and pro-inflammatory agents, allowing adaptation and survival under stress conditions. Keap1 is equipped with reactive cysteine residues that act as sensors for endogenously produced and exogenously encountered small molecules (termed inducers), which have a characteristic chemical signature, reactivity with sulfhydryl groups. Inducers modify the cysteine sensors of Keap1 and impair its ability to target Nrf2 for ubiquitination and degradation. Consequently, Nrf2 accumulates, enters the nucleus and drives the transcription of its target genes, which encode a large network of cytoprotective proteins. Here we summarize the early studies leading to the prediction of the existence of Keap1, followed by the discovery of Keap1 as the main negative regulator of Nrf2. We then describe the available structural information on Keap1, its assembly with Cullin3, and its interaction with Nrf2. We also discuss the multiple cysteine sensors of Keap1 that allow for detection of a wide range of endogenous and environmental inducers, and provide fine-tuning and tight control of the Keap1/Nrf2 stress-sensing response.

## 1 Introduction

Mammalian cells have evolved elaborate mechanisms for protection against environmental challenges, including those caused by exposure to oxidants, electrophiles and pro-inflammatory agents, which are involved in the pathogenesis of almost all chronic disease and ageing. Deficiencies in these protective mechanisms are associated with increased disease risk and accelerated disease progression. These systems can be upregulated by various small molecules, termed inducers. Many of them are synthesized in plants, including edible plants, and are thus present in the human diet. Indeed, the majority of the health-promoting properties of diets rich in fruits and vegetables have been attributed to specific phytochemicals, which are able to induce defence systems in mammalian cells. Induction of these natural defences is protective against damage and allows adaptation and survival under conditions of stress.

One such defence system comprises a network of cytoprotective genes, the expression of which is regulated by transcription factor NF-E2 p45-related factor 2 (Nrf2, gene name *NFE2L2*) [1]. The battery of Nrf2-regulated proteins encompasses a large number of diverse detoxification, antioxidant, and anti-inflammatory proteins, as well as enzymes with essential roles in cell metabolism, placing Nrf2 at the interface of redox and intermediary metabolism [2]. Under homeostatic conditions, Nrf2 is a short-lived protein that is continuously targeted for ubiquitination and proteasomal degradation. Depending on the specific conditions, such as the presence or absence of growth factors or endoplasmic reticulum (ER) stress, the process of Nrf2 degradation is mediated by several ubiquitin ligase systems, including Kelch-like ECH associated protein 1 (Keap1) [3], a substrate adaptor protein for Cullin3-based Cullin-RING E3 ubiquitin ligase [4], [5], [6], β-transducin repeat-containing protein (β-TrCP), a substrate adaptor for Skp1-Cullin1-based Cullin-RING E3 ubiquitin ligase [7], [8], and the E3 ubiquitin ligase Hrd1 [9]. This review focuses on Keap1, which functions as a substrate adaptor protein for the degradation of Nrf2 and serves as an intracellular sensor for inducers, by utilising a number of reactive cysteine residues.

### 1.1 Discovery of Keap1: historical perspective

In the early 1960s, Charles Huggins found that pretreatment with small doses of 7,12-dimethylbenz[*a*]anthracene, other polycyclic aromatic hydrocarbons, or of aromatic amines protects against the toxicity and carcinogenicity of a high dose of the same damaging agent [10], [11]. In the 1970s, Lee Wattenberg showed that similar protection can be achieved by dietary constituents, including phytochemicals such as indole-3-carbinol, a compound present in cruciferous vegetables, as well as phenolic antioxidants, such as 2(3)-*tert*-butyl-4-hydroxyanisole (BHA), a commonly added preservative in processed food [12]. However, the underlying mechanism of this protection remained elusive until the studies of Paul Talalay and Ernest Bueding. They showed that such agents are able to induce phase 2 drug metabolising enzymes, which in turn lead to more efficient detoxification and excretion of the pro-carcinogens [13], [14]. This phenomenon was termed “the phase 2 response” [15], or the “electrophile counterattack response” [16].

**Fig. 1:**
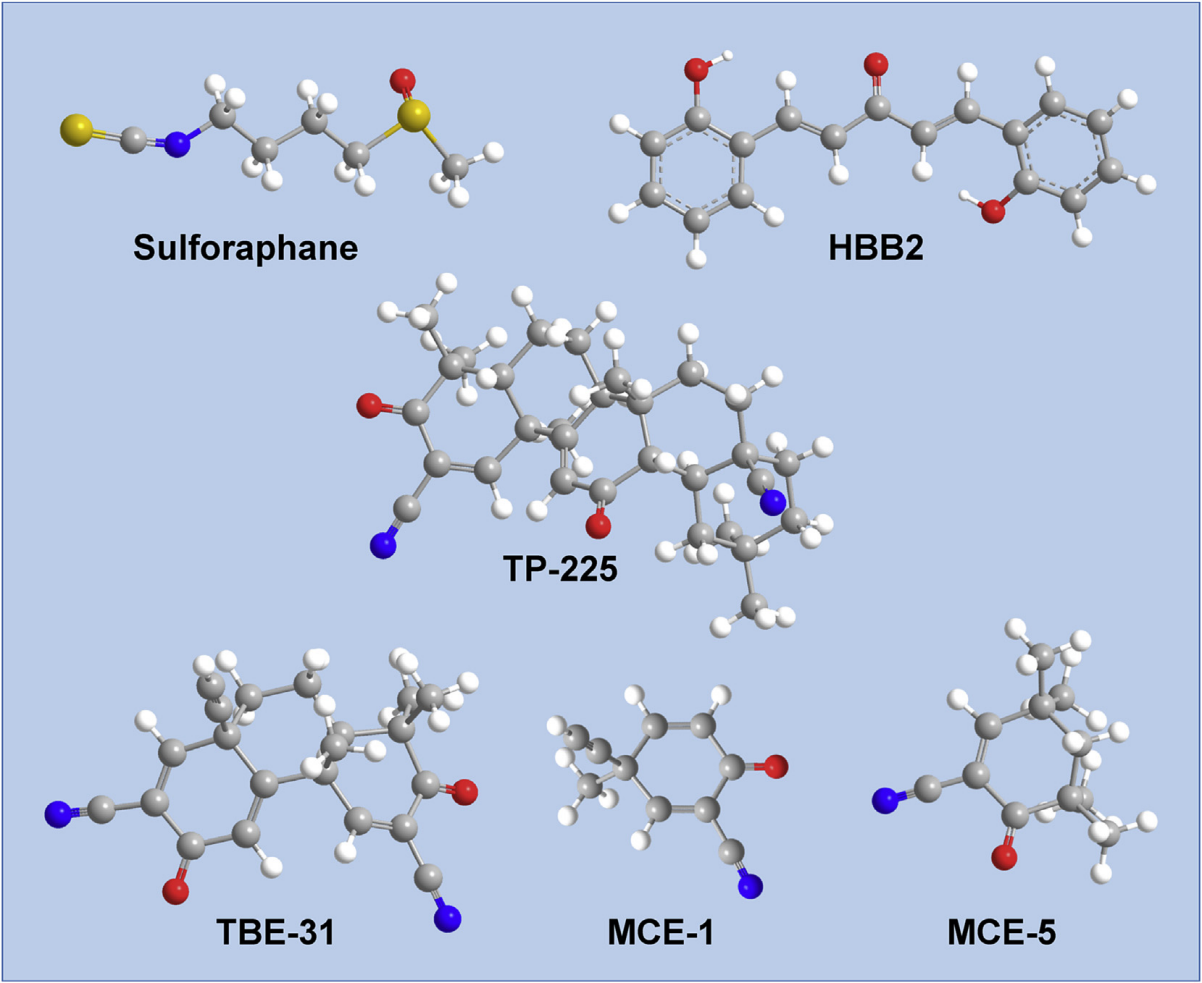
Examples of small-molecule Nrf2 activators that react with cysteine sensors of Keap1. Shown are model structures of the isothiocyanate sulforaphane [1-isothiocyanato-4*R*-(methylsulfinyl)butane], the double Michael acceptor HBB2 [bis(2-hydroxybenzylidene)acetone], and the cyanoenones TP-225 [2-cyano-3,12-dioxooleana-1,9(11)-dien-28-onitrile], TBE-31 [(±)-(4b*S*,8a*R*,10a*S*)-10a-ethynyl-4b,8,8-trimethyl-3,7-dioxo-3.4b,7,8,8a,9,10,10a-octahydrophenanthrene-2,6-dicarbonitrile], MCE-1 [3-ethynyl-3-methyl-6-oxocyclohexa-1,4-dienecarbonitrile] and MCE-5 [3,3,5,5-tetramethyl-6-oxocyclohex-1-enecarbonitrile].

Because activation of the phase 2 response had protective effects in various animal models of human cancer, a quantitative cell culture-based bioassay was developed to allow for rapid screening for potential activators, using NAD(P)H:quinone acceptor oxidoreductase 1 (NQO1) as a prototypic marker enzyme of the phase 2 response [17]. This assay, which became known as ‘the Prochaska bioassay’ [18] is still widely and reliably used today to identify and rank the inducer potencies of activators of Nrf2, including pure compounds as well as complex mixtures, such as plant extracts. Its application as an activity-guided fractionation led to the isolation of the isothiocyanate sulforaphane [1-isothiocyanato-4*R*-(methylsulfinyl)butane] (Fig. 1) as the principal NQO1 inducer in broccoli [19]. Sulforaphane remains one of the most potent naturally occurring Nrf2 activators known to date. It has shown beneficial effects in numerous animal models of human disease, and in clinical trials [20], [21], [22], [23], [24], [25], [26], [27].

Initial screens using the Prochaska cell culture bioassay as well as rodent models revealed a bewildering array of structurally diverse compounds able to induce NQO1 [28], [29]. However, in spite of this diversity, they all had a common chemical signature, that of sulfhydryl reactivity. In a seminal paper published in 1988 [30], Paul Talalay and his colleagues stated: ‘ … *the capacity of an extraordinary variety of seemingly unrelated anticarcinogens to induce protective enzymes can be attributed to the presence, or acquisition by metabolism, of a simple and hitherto unrecognized chemical property: that of a Michael reaction acceptor* … ’, and further predicted the existence in the cell of ‘ … *a protein endowed with highly reactive cysteine residue(s) that serves as the sensor for small-molecule inducers of the phase 2 response*’. These were courageous statements, which were not readily embraced by the scientific community; this was the era of ‘ligand: receptor’ interactions, and chemical reactivity was yet to be recognized as a mechanism for sensing environmental signals.

Given these early insights, when Masayuki Yamamoto and his colleagues discovered Keap1 as the main negative regulator of Nrf2 and the expression of its downstream target genes [3], it was logical to ask whether Keap1 contained reactive cysteine residues. Examination of its primary structure showed that Keap1 is a cysteine-rich protein, containing 25 and 27 cysteine residues within the mouse and the human homologues, respectively. Importantly, 10 of these cysteines are adjacent to positively-charged amino acids; such proximity is known to decrease the pKa of the neighbouring cysteine sulfhydryl group and thus stabilize the thiolate anion, maintaining the cysteine in a reactive state [31]. Because it negatively regulates Nrf2 and contains reactive cysteine residues, Keap1 became the perfect candidate for the inducer sensor. Indeed, during the subsequent years, a number of different laboratories have identified cysteine modifications of Keap1 by numerous inducers [reviewed in Refs. [32], [33], [34]]. It is now widely accepted that Keap1 is the cysteine-based mammalian intracellular sensor for exogenous and endogenous electrophiles and oxidants, and that cysteine modifications of Keap1 lead to Nrf2 accumulation, nuclear translocation, and transcriptional upregulation of Nrf2-dependent cytoprotective genes.

### 1.2 Structure of Keap1

The Keap1 protein was first identified as a binding partner of Nrf2 and named Keap1 based on structural similarities with the *Drosophila* Kelch protein [3]. Keap1 functions as the substrate-recognition module of a Cullin-RING ligase (CRL) E3 ligase complex constructed around the Cullin3 scaffold protein. CRLs formed around Cullin3 are unusual for two reasons. Firstly, in most CRLs the substrate recruitment protein associates with the N-terminus of the Cullin scaffold protein via an adapter protein. For example the well-characterized SCF complex (Skp, Cullin, F-box containing complex), uses the adapter protein Skp1 to connect Cullin1 to the substrate-recognition module, an F-box containing protein such as Skp2 [35]. Cullin3-based E3 ligases use a single protein to act as both the adapter and substrate-recognition protein. Secondly, the Cullin3-CRLs function as homodimers. This feature is key to their mechanism and so far unique among the CRLs [36], [37], [38], [39], [40].

Keap1 is a member of the BTB-Kelch family of proteins. It has retained the gene name *KEAP1*, but is also known as KLHL19 to conform to the naming convention of the protein family. BTB-Kelch proteins are divided into KLHL proteins and KBTB proteins. KLHL proteins typically consist of an N-terminal Broad complex, Tramtrack, and Bric à brac (BTB) domain, a BACK domain, and a C-terminal Kelch domain made up of 5–6 Kelch motif repeats. KBTB proteins consist of an N-terminal BTB domain and C-terminal Kelch domain, made up of 2–4 Kelch repeats. They occasionally also have a BACK domain.

**Fig. 2:**
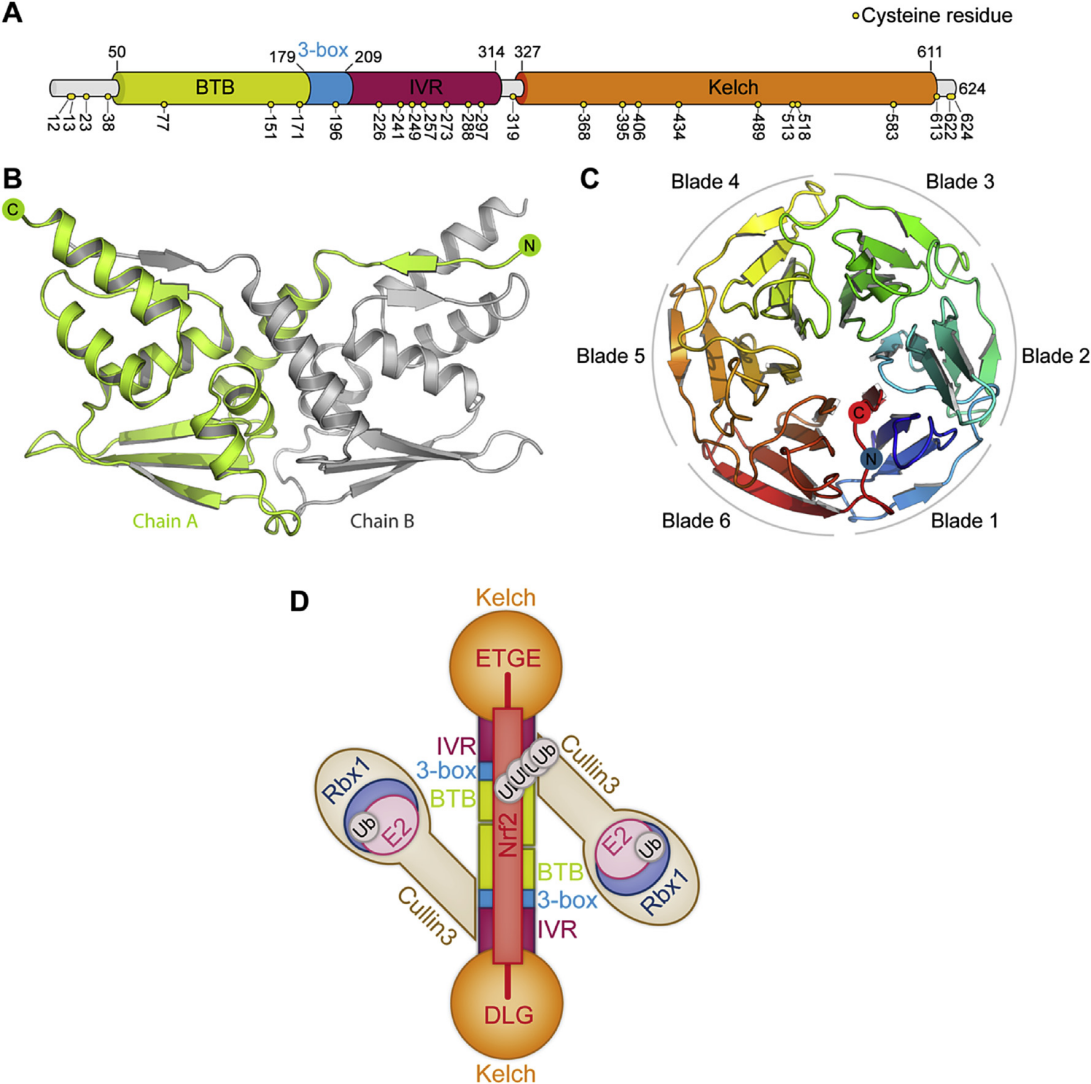
**(A)** Domain organization of human Keap1. The locations of the 27 cysteine residues are indicated. **(B)** The crystal structure of the Keap1 BTB domain (PDB ID: 4CXI). One monomer is coloured as in **(A)** and the neighbouring monomer coloured grey to highlight the dimer interface. **(C)** Crystal structure of the Keap1 Kelch domain (PBD ID: 2FLU), coloured blue to red from N- to C-terminus. The 6 ‘blades’ of the propeller are indicated and numbered. **(D)** Cartoon representation of the complete Keap1/Cullin3 E3 ligase complex. The Keap1 dimer engages a single molecule of Nrf2 via the ETGE and DLG motifs and associates with Cullin3, which engages a RING protein such as Rbx1 with the C-terminal domain. Rbx1 recruits an activated E2, which is then able to polyubiquitinate the bound Nrf2, resulting in Nrf2 degradation. (For interpretation of the references to colour in this figure legend, the reader is referred to the web version of this article.)

As a member of the KLHL family, Keap1 is composed of an N-terminal region (residues 1–49), a BTB domain (residues 50–179) and a C-terminal Kelch domain (residues 327–611) with an intervening BACK domain that is most commonly referred to as the intervening region (IVR, residues 180–314) (Fig. 2A). There is currently no crystal structure of the full-length Keap1 protein, but crystal structures of individual domains and family members have provided valuable information about the structure of Keap1.

The BTB domain is named after the *Drosophila* proteins Broad complex, Tramtrack, and Bric à Brac [41]. It is also known as a POZ domain [42]. The BTB domain is a protein-protein interaction domain, shown to form hetero- and homo-dimers *in vitro* [42]. In Keap1, as in other BTB-Kelch proteins, the BTB domain mediates homodimer formation. The first crystal structure of a BTB domain to be solved was from the human protein PLZF [43]. This crystal structure revealed a highly-symmetrical dimer interface occupying roughly a quarter of the domain surface area. An N-terminal β-sheet of one monomer forms a two-stranded β-sheet with a β-strand from the main body of the neighboring monomer. The structures of a number of other BTB domains have shown that this interface is well conserved, including the BTB domain of Keap1, the structure of which was recently reported (Fig. 2B) [44].

The BACK domain was originally defined as a conserved region occurring in proteins with BTB domains and C-terminal Kelch repeats [45]. Sequence analysis predicted that the domain would be entirely helical. The structure of the Keap1 IVR (a BACK domain) has yet to be solved, but crystal structures of the BACK domains of KLHL3 and KLHL11 are available [46], [47]. They confirm that the domain is entirely helical and show that the domain is highly extended, presumably to position the Kelch domain correctly for substrate recruitment.

At the N-terminal end of the IVR is a bihelical motif known as the ‘3-box’ [39]. The 3-box is a crucial structural element required for the interaction between Keap1 and Cullin3. The BTB domain with a C-terminal 3-box are sufficient for BTB-Cullin3 assembly, but in experiments using the BTB-MATH protein SPOP, the deletion of the 3-box resulted in more than a 10-fold drop in the affinity of SPOP for Cullin3 [39].

The Kelch domain is located at the C-terminus of Keap1. Numerous crystal structures of the Keap1 Kelch domain have now been solved, driven in part by an interest in targeting the domain with small molecule inhibitors [48], [49], [50], [51], [52], [53], [54], [55], [56], [57]. The domain is made up of 6 Kelch repeats, each of which forms one of the ‘blades’ of a highly-symmetrical 6-bladed β-propeller, with a narrow solvent-filled channel at the center. Each blade is a four-stranded antiparallel twisted β-sheet. The propeller is closed via a ‘C-terminal strand closure’ mechanism: three of the strands that make up blade 1 are from the N-terminal end of the sequence, while the fourth is from the C-terminal end. This forms a stable interface between blades 1 and 6 (Fig. 2C). Structures of additional Kelch domains have shown that this blade-closure arrangement is a common feature of Kelch domains [47], [58]. At the top of the domain is a shallow, positively-charged recess that functions as the substrate-binding pocket.

Keap1 homodimerizes via the BTB domain, employing an interface very similar to that of the PLZF BTB domain, despite limited sequence identity between the two domains [43], [44]. A CryoEM reconstruction of the mouse Keap1 protein revealed it to be a highly-symmetric dimer resembling a ‘pair of cherries’ [40]. The two Kelch domains are joined by a ‘stem’ of the BTB and IVR regions, positioning the two substrate binding pockets approximately 95 Å apart. The symmetry axis is the BTB dimer interface.

### 1.3 Assembly of Keap1 with Cullin3

Keap1 associates with the N-terminal domain of Cullin3 via the BTB domain and 3-box. Crystal structures of BTB proteins in complex with Cullin3 have shown the interface in detail [46], [47], [59]. Similar to other Cullin proteins, the N-terminal domain of Cullin3 is composed of three ‘Cullin repeats’. Each repeat consists of a five-helix bundle motif. The N-terminal end of this domain interacts with the BTB domain, 3-box and IVR of Keap1, projecting away from the ‘stem’ of the Keap1 dimer at roughly a 45° angle. Helices α2 and α5 pack up against the surface of Keap1. An 8-residue strand N-terminal to helix α1 extends across the width of the domain, running along the top of the 3-box and contacting the BTB and IVR domains. In studies with Cullin3 and KLHL11, deletion of the residues N-terminal to the α1 helix resulted in over a 30-fold reduction in affinity [47]. This is a feature crucial to the Cullin3-BTB interaction, presumably necessary to compensate for the lack of Skp1-type adapter proteins in Cullin3 based CRLs. The Cullin3 C-terminal domain supports a RING protein such as Rbx1, which is required to recruit an activated E2 ubiquitin-conjugating enzyme. No crystal structures have yet been solved of full-length Cullin3, but crystal structures have been solved of several CRL complexes constructed around full-length Cullins and isolated Cullin C-terminal domains in complex with RING proteins such as Rbx1, showing the general structure of full-length Cullin proteins and the Cullin-RING interface [35], [60], [61], [62], [63], [64], [65]. The Cullin C-terminal domain is a globular domain that engages a RING protein via a 5-stranded β-sheet in which the second strand is the N-terminal β-sheet of the associated RING protein [35], [60], [61], [62], [63], [64], [65]. Experiments using size-exclusion chromatography and analytical ultracentrifugation to study a recombinantly-expressed Keap1/Cullin3/Rbx1 complex showed that two molecules of Keap1 assemble with one molecule of Cullin3 and Rbx1, a 2:1 stoichiometry [66]. However, experiments using the BTB protein SPOP in complex with Cullin3 found that the two proteins assemble with a 2:2 stoichiometry [39], and an *in vitro* assay conducted using Keap1:Cullin3 and SPOP:Cullin3 complexes suggested that both complexes assemble with a 2:2 stoichiometry [67]. Additionally, multiple crystal structures have now been solved of BTB proteins in complex with Cullin3 that all show a 2:2 stoichiometry [46], [47], [59]. This evidence suggests that Cullin3 binds to Keap1 with 2:2 stoichiometry, and consequently the complex forms a homodimer with the BTB domain of Keap1 functioning as the interface (Fig. 2D).

### 1.4 The Keap1/Nrf2 interaction

The dimeric architecture of the complex is central to its function. Two molecules of Keap1 engage a single molecule of the substrate, Nrf2. Each of the Kelch domains binds to one of two motifs in the Nrf2 protein, known as the ETGE and DLG motifs [37]. These two motifs are situated at either side of a central lysine-rich α-helix, so that binding to Keap1 positions the central helix in the middle of the complex ready for the attachment of ubiquitins from the activated E2 enzymes on either side (Fig. 2D). It is thought that Nrf2 interfaces with Keap1 via a proposed ‘hinge-and-latch’ mechanism [52]. The ETGE motif folds into a β-turn and inserts into the substrate binding pocket of the Keap1 Kelch domain, forming specific electrostatic interactions between the sidechains of E79 and E82 of Nrf2 and several Keap1 residues lining the substrate binding [50], [51]. The DLG motif binds to the opposite Kelch domain in a similar binding mode to the ETGE motif but with an affinity 200-fold lower [37], [55]. Thermodynamic analysis revealed that the ETGE motif binds with a much slower on/off rate than the DTG motif, but both motifs are necessary for activity [36], [55]. In this way the ETGE is thought to function as the ‘hinge’ and the DTG motif as the ‘latch’, positioning the Nrf2 lysine-rich helix for ubiquitination [68].

**Fig. 3:**
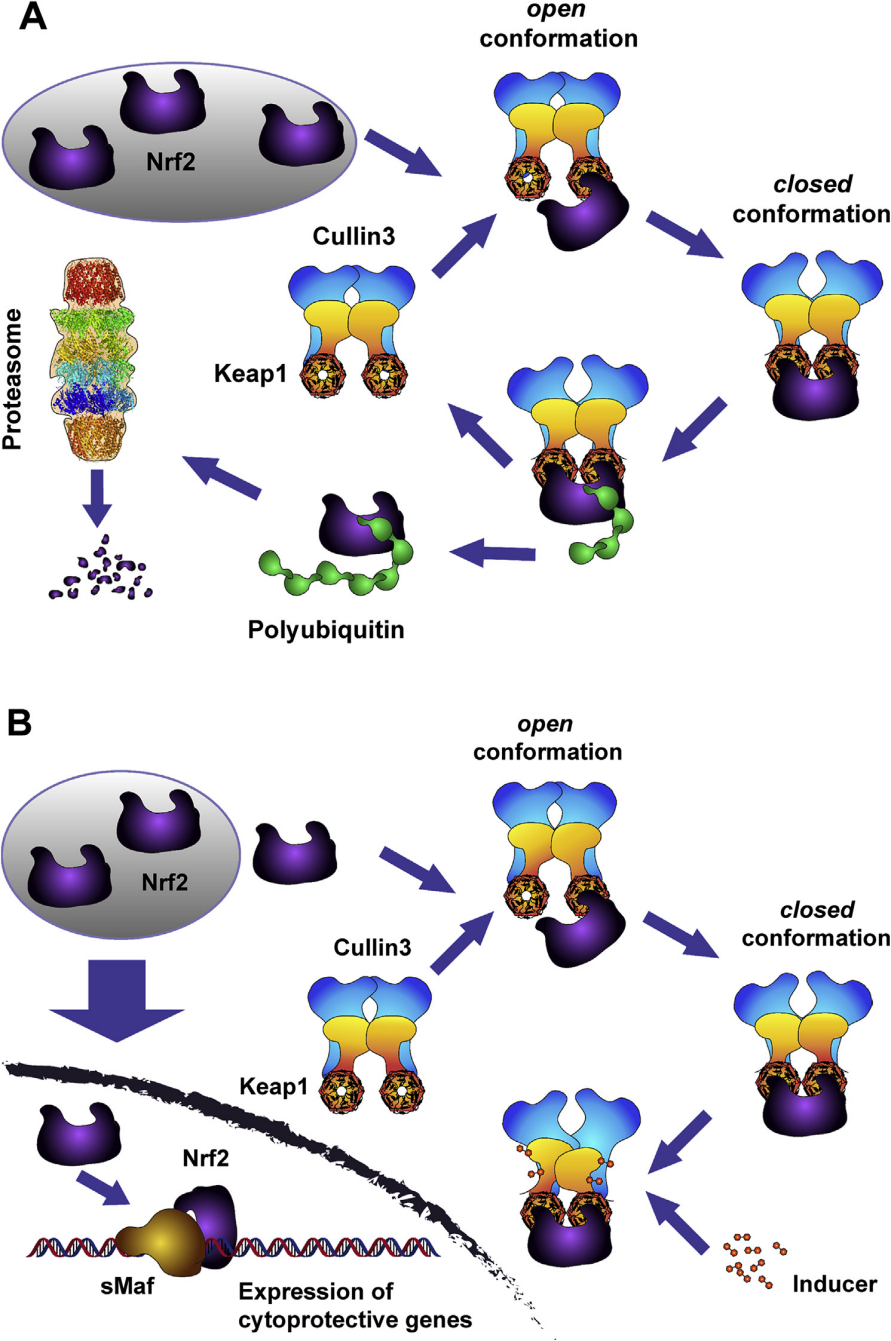
The cycle of Keap1-mediated degradation of Nrf2. **(A)** At homeostatic conditions, Keap1 uses a cyclical mechanism to target Nrf2 for ubiquitination and degradation, whereby the Neh2 domain of *de novo* synthesized Nrf2 binds sequentially to the Kelch domains of the Keap1 dimer, first through its high affinity “ETGE” binding motif to form the *open* conformation of the Keap1: Nrf2 protein complex, followed by the low affinity “DLG” binding motif to form the *closed* conformation. In the *closed* conformation of the protein complex, Nrf2 is ubiquitinated and subsequently degraded through the proteasome. Free Keap1 is regenerated, allowing the cycle to start again. **(B)** Electrophiles and oxidants (inducers) block the cycle of Keap1-mediated degradation of Nrf2 by chemically modifying cysteine sensors of Keap1 and disabling its substrate adaptor function, leading to accumulation of the protein complex in the *closed* conformation. As a result, Nrf2 is not degraded, and Keap1 is not regenerated. This allows *de novo* synthesized Nrf2 to accumulate, translocate to the nucleus, heterodimerize with a small Maf transcription factor (sMaf), and initiate transcription of downstream target genes.

Under basal conditions, the dimeric Keap1 CRL complex engages an Nrf2 molecule. Covalent attachment of a Nedd8 protein to a highly-conserved lysine in the Cullin3 C-terminal domain leads to a reconfiguration of the complex that optimally positions the associated E2 enzymes above the bound Nrf2, leading to its polyubiquitination and subsequent degradation by the proteasome [47], [62], [69]. Exposure to electrophiles or reactive oxygen species causes the cessation of polyubiqutination. The reactive ‘sensor’ cysteine residues in Keap1 are modified. The ‘hinge-and-latch’ model states that this reduces the affinity of Keap1 for Nrf2 but does not lead to release. Instead, newly-synthesized Nrf2 is translocated to the nucleus, triggering the transcription of Nrf2-dependent genes [52], [68]. More recently, an alternative model was proposed known as the ‘conformation cycling’ model. This model posits that Keap1 uses a cyclic mechanism to target Nrf2 for ubiquitination and proteasomal degradation (Fig. 3A) [70], [71]. An important feature of this cyclic mechanism is that it ensures regeneration of Keap1, which allows the cycle to proceed. Inducers bind and chemically modify specific reactive cysteine residues of Keap1, or directly disrupt the protein: protein interaction between Keap1 and Nrf2, thus blocking the cycle of Keap1-dependent Nrf2 degradation. This block allows *de novo* synthesized Nrf2 to accumulate, translocate to the nucleus, and initiate transcription of Nrf2-dependent cytoprotective genes (Fig. 3B).

### 1.5 The cysteine sensors of Keap1: lessons from experiments with purified recombinant Keap1

**Fig. 4:**
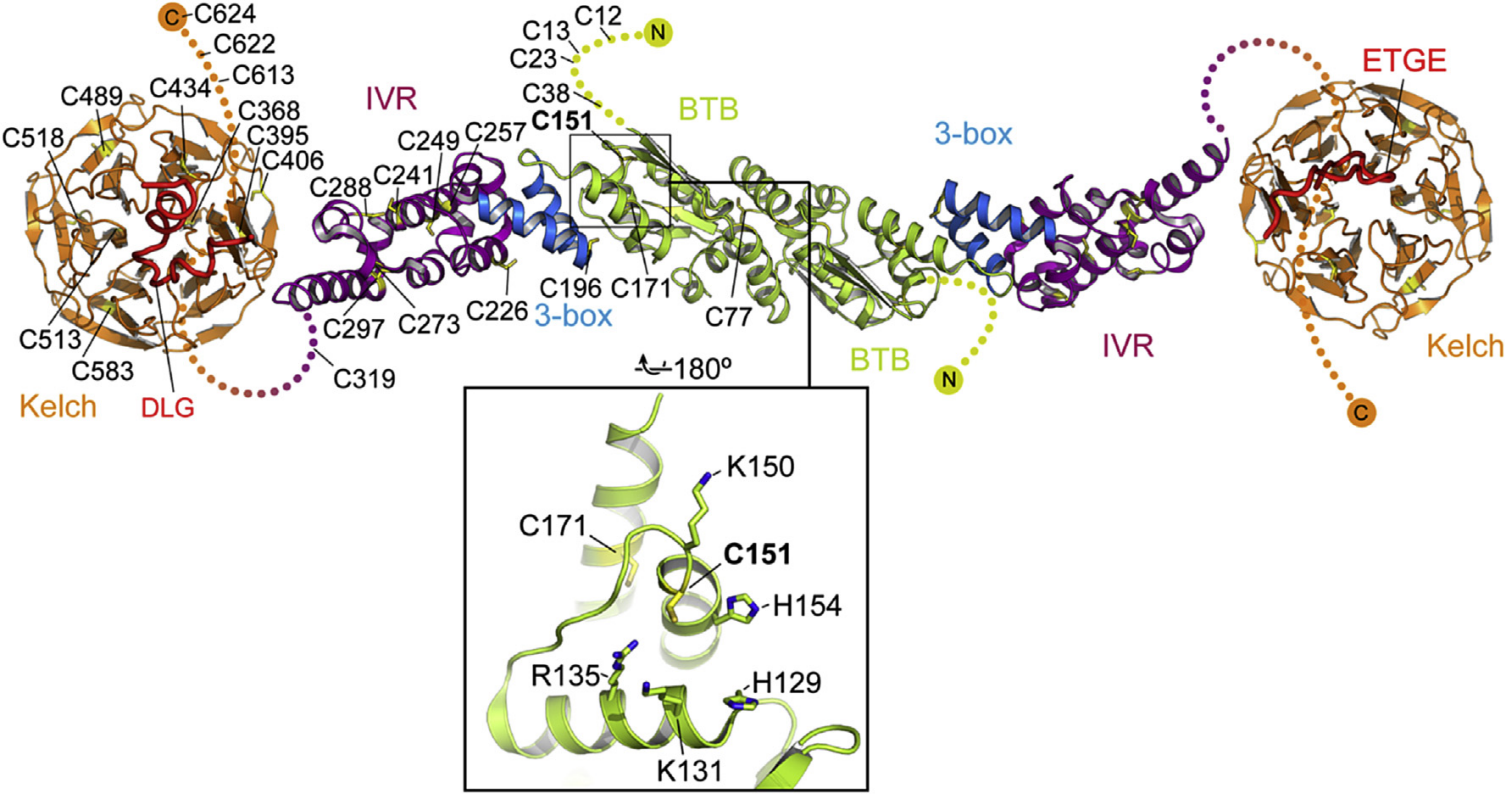
Model of the complete Keap1 structure, using the structure of the KLHL11 BTB and BACK domains (PDB ID: 3I3N) as the BTB and IVR domains and the Keap1 Kelch domain in complex with either the ETGE motif (PDB ID: 2FLU) or the DLG motif (PDB ID: 3WN7). Domains are coloured as in Fig. 2A. The Keap1 cysteine residues have been modelled onto the KLHL11 structure and numbered as in the human Keap1 sequence. The inset panel shows the environment of the highly-reactive C151 (PDB ID: 4CXI), with the surrounding basic residues shown as sticks and numbered.

The discovery of Keap1 as a negative regulator of Nrf2 made it possible to test the idea that cysteine residues of Keap1 serve as the sensors for inducers. As mentioned above, Keap1 is a cysteine-rich protein (Fig. 4), and the 27 cysteine residues in the human protein are all reactive to varying degrees [72]. Initial experiments employed ultraviolet-visible (UV-VIS) spectrophotometry and compared the spectral changes of various inducers upon addition of purified recombinant Keap1. In this way, it was shown that Keap1 binds directly to inducers of three different classes, i.e., the isothiocyanate sulforaphane, the double Michael acceptor bis(2-hydroxybenzylidene)acetone, as well as a number of cyanoenones: the pentacyclic TP-225, the tricyclic TBE-31, and the monocyclic MCE-1 and MCE-5 [73], [74], [75] (Fig. 1). Notably, although such inducers bind to Keap1 covalently, the reaction is readily reversible [76], which makes them suitable for chronic *in vivo* administration [77].

A mass-spectrometry approach to detect covalent adducts in Keap1 required incubation with an inducer that binds to cysteines irreversibly, and the steroid dexamethasone 21-mesylate (Dex-mes) was initially used. This led to identification of four cysteine residues (C257, C273, C288 and C297) within the IVR domain, and a fifth one, C613 in the C-terminal region of murine Keap1, all of which were modified when purified recombinant murine Keap1 was incubated with Dex-mes [73]. Incubation of human Keap1 with two separate biotin-tagged electrophiles, *N*-iodoacetyl-*N*-biotinylhexylenediamine (IAB) or 1-biotinamido-4-(4‘-[maleimidoethylcyclohexane]carboxamido)butane (BMCC), led to cysteine modifications of Keap1, but in each case, the spectrum of the modified cysteines was different, as analyzed by liquid chromatography-tandem mass spectrometry. Thus, Eggler et al. [78] reported that IAB alkylated most readily C151, C288, and C297, followed by C319, C257, C273, and C613. Using somewhat different reaction conditions, Hong et al. [79] showed that IAB modified primarily the IVR cysteines C196, C226, C241, C257, C288, as well as C319, whereas BMCC reacted with C196 and C249 in the IVR, C77 in the BTB domain, and with C368 and C489 in the Kelch domain.

Unlike the stable alkylation adducts formed by Dex-mes, IAB and BMCC, binding of the dietary isothiocyanate sulforaphane to Keap1 leads to the formation of unstable thionoacyl adducts, which are labile to hydrolysis and transacylation reactions. To allow for detection of these adducts, a liquid chromatography-tandem mass spectrometry method was developed and further optimised to minimize adduct decomposition [80]. It was found that sulforaphane modified cysteine residues in purified recombinant human Keap1, mainly in the Kelch domain. The most consistently modified cysteine was C489, followed by C513, C518, and C583. Adducts with the IVR cysteines C226 and C249, as well as with C77 located in the BTB domain, and C624 in the C-terminal region, were also detected. A subsequent study showed that by modifying the conditions for incubation and sample processing (eliminating the iodoacetamide treatment step and thus reducing the possibility for competition with sulforaphane for the formation of reversible sulforaphane-cysteine adducts, as well as shortening the sample preparation time), C151 could be also detected as one of the four (C38, C151, C368 and C489) most readily modified cysteine residues in Keap1 by sulforaphane [81]. In addition to sulforaphane, C151 is also the most reactive cysteine in Keap1 towards three other electrophilic natural products, xanthohumol, isoliquiritigenin, and 10-shogaol, all of which activate Nrf2 [82].

Together, the experiments with purified recombinant Keap1 established that cysteine residues in Keap1 serve as sensors for a variety of inducers. However, depending on the reaction conditions and the type of the inducer, multiple cysteine residues could be modified. It was next important to determine which of these cysteine residues function as inducer sensors in the context of the cellular environment. This question was addressed by employing both genetic and pharmacologic means. Due to its low abundance, the next set of experiments required ectopic expression of Keap1 in cells.

### 1.6 The cysteine sensors of Keap1: lessons from experiments with ectopically expressed Keap1 and its mutants in mammalian cells

The use of ectopically-expressed Keap1 or of Keap1 carrying mutations in some of the identified inducer-modified cysteine residues revealed that substitution of C273 or C288 with either serine or alanine (i.e., C237A/S and C288A/S) rendered Keap1 unable to repress Nrf2 activity under homeostatic conditions in cells [83], [84], [85]. This finding was further confirmed *in vivo* by generating transgenic mice expressing either C273A or C288A Keap1 mutants [86]. The inability of these Keap1 mutants to repress Nrf2 correlated with reduced ubiquitination of Nrf2, but did not affect binding of Keap1 to Nrf2 or Cullin3 [5], [6], [87], suggesting that modification of C273 and C288 by inducers could decrease the rate of ubiquitination and degradation of Nrf2. A recent study systematically introduced amino acid substitutions of C273 and C288, and found that C273W and C288N mutations did not affect the ability of Keap1 to repress Nrf2 [88]. This finding allowed generation of stable mouse embryonic fibroblast (MEF) cell lines for testing of the functional importance of C273 and C288 in inducer sensing, which previously had been not been possible due to the loss-of-function phenotype of the C273A/S and C288A/S mutants.

In stark contrast with the loss-of-function phenotype of the C237A/S and C288A/S mutants of Keap1, mutation of C151 to serine rendered Keap1 a constitutive repressor of Nrf2 at both homeostatic and induced states in cells [83] and in transgenic mice [86]. C151 was found to be indispensable for sensing the classical inducers sulforaphane and *tert*-butyl hydroquinone (tBHQ), but not for sensing other inducers such as the environmental toxin arsenite [89] or the endogenous cyclopentene prostaglandin 15-deoxy-Δ12,14-prostaglandin J_2_, (15-dPGJ_2_), which is dependent on C273 [90] or C288 [88]. Together, these findings started to shed light on the existence of a degree of specificity, i.e. that specific cysteine residues in Keap1 may form discrete sensors, which ‘respond’ to certain types of inducers. This possibility was addressed by McMahon and colleagues [91]. Using ectopically expressed murine Keap1 in mammalian cells, these researchers found that C151 and C288 formed the basis for two discrete cysteine sensors. In addition, a third sensor, termed ‘zinc sensor’, is formed by H225, C226 and C613. Each of the three sensors shows specificity for certain inducers (see below). This complexity of responses has given rise to the idea of a ‘cysteine code’, the hypothesis that different inducers modify specific combinations of cysteines in order to tightly control the Keap1/Nrf2 stress-sensing response [32], [92], [93], [94].

It is now clear that C151 is required for sensing of nitric oxide, sulforaphane, tBHQ, diethylmaleate, dimethylfumarate, and CDDO-Im [83], [88], [91], [95]. C288 responds to the cyclopentenone prostaglandins 15-dPGJ_2_ and prostaglandin A_2_ (PGA_2_), the electrophilic nitro-oleic acid (OA-NO_2_) [88], the alkenals acrolein and 4-hydoxynonenal [91], and daillyl trisulfide, a volatile component of garlic oil [96]. H225, C226 and C613 comprise the sensor for Zn^2+^, Cd^2+^, Se^4+^, As^3+^ [91], and H_2_S [97]. The ability of Keap1 to sense Zn^2+^ is consistent with a previous report, which found that recombinant Keap1 binds Zn^2+^ stoichiometrically, with an association constant of 1011 M^−1^ [98]. A study with sulfoxythiocarbamate alkyne (STCA), an analog of sulforaphane, in which the isothiocyanate group is replaced by a sulfoxythiocarbamate group that forms stable thiocarbamate adducts with cysteine residues, identified C273, C288 and C613 of ectopically expressed Keap1 as being modified by this mildly electrophilic inducer [99]. Inhibition of Keap1 by H_2_S was found to be facilitated by the formation of an intramolecular disulfide bridge between C226 and C613 [97].

Notably, among the sensor cysteines of Keap1, C151 is best characterized. Evidence from a number of studies has suggested that C151 is the most reactive and critical to the Keap1/Nrf2 stress-sensing response [6], [78], [83]. As described above, C151 has been identified as the most frequently modified by Nrf2-activating agents in mass spectrometry studies [82], [100]. C151 is located in the BTB domain, at the N-terminal end of the α5 helix, and is thought to be highly reactive as a result of its environment, as it is surrounded by basic residues (H129, K131, R135, K150, and H154), which lower the pK_a_ of C151, enabling it to exist as the thiolate anion at physiological pH (Fig. 4). Mutagenesis studies showed that exchanging many of these residues for methionine results in a significant reduction in C151 reactivity [91]. A model was proposed suggesting that covalent modification of C151 caused a dissociation of the Keap1/Cullin3 heterodimer, consequently blocking Nrf2 ubiquitination [101], [102]. However, a crystal structure of the Keap1 C151W mutant BTB domain, designed to mimic a modified C151, showed no conformational changes that would impact Cullin3 binding [44], and data from live cells showed no evidence that the mechanism involves a Keap1/Cullin3 dissociation [70], [103]. An alternative model suggests that modification of the sensor cysteines triggers a conformational change in Keap1 but not the dissociation of Nrf2 or Cullin3 [98], [104]. This is supported by experiments using a hydrophobicity probe, which showed that the hydrophobicity of recombinant Keap1 (determined by the intrinsic tryptophan fluorescence) decreases upon addition of the cysteine-reactive inducers 4,4’-dipyridyl disulfide and sulforaphane [98]. However, the exact nature of this conformational change is still unclear.

Although less extensively studied, two other cysteines located in the Kelch domain, i.e. C434 and C368, are also reactive. Both of these residues were shown to be modified by glutathione, and molecular modeling has indicated that the consequences of such modification would alter the conformation of the Kelch domain in such a way as to block the Keap1/Nrf2 interaction [72]. In addition, C434 was shown to be modified by a nitrated derivative of cGMP, 8-nitroguanosine 3′,5'-cyclic monophosphate (8-nitro-cGMP), leading to Nrf2 activation [105]. Overall, it is now clear that Keap1 utilizes multiple cysteine residues as sensors for detection of a wide range of endogenous and environmental inducers.

### 1.7 The fate of Keap1 after sensor cysteine modification

To address the fate of Keap1 after modification of its sensor cysteine(s), Hong et al. [79] used ectopically expressed FLAG-Keap1 in HEK293 cells. Treatment with IAB or tBHQ led to formation of high molecular weight Keap1 species, which were identified as K-48-linked polyubiquitin conjugates by immunoblotting and liquid chromatography tandem mass spectrometry. Coincidentally with Keap1 polyubiquitination, Nrf2 stabilization and nuclear accumulation were observed. A model was proposed according to which Keap1 cysteine modification by electrophiles triggers a switch of Cullin3-dependent ubiquitination from Nrf2 to Keap1, ultimately leading to Nrf2 activation. More recently, Taguchi et al. [106] proposed a different model. These authors showed that under conditions of autophagy deficiency (i.e. in livers of *Atg7*-or *p62*-deficient mice), the levels of Keap1 are increased. Nutrient starvation in human hepatoma (HepG2) cells caused a decrease in the levels of Keap1. Together, these results suggest that Keap1 is degraded through autophagy in a p62-dependent manner. Exposure to electrophiles, such as tBHQ, shortens the half-life of Keap1. The authors proposed that autophagy-mediated accelerated turnover of Keap1 contributes to the recovery of the Keap1 activity after modification of its sensor cysteine(s) by electrophiles.

Some of the most potent electrophilic inducers, such as the isothiocyanates and the cyanoenones induce cytoprotective responses in the order of their electron affinity [107] and bind to cysteine in a reversible manner [76], and may cause formation of disulfide bond(s) in Keap1. It is therefore possible that such cysteine modification(s) of Keap1 can be reversed by the concomitant increases in the intracellular levels of glutathione and thioredoxin/thioredoxin reductase systems, both of which are Nrf2 transcriptional targets. This notion is supported by the finding that simultaneous inactivation of the glutathione (by chemical inhibition) and thioredoxin (by shRNA-mediated knockdown of thioredoxin reductase 1) systems leads to constitutive Keap1 oxidation [108]. Thus, it is possible that in contrast to irreversibly modified Keap1 which is destroyed by ubiquitination/proteasomal degradation and/or autophagy and needs to be replenished by *de novo* synthesis, reversible modification(s) of Keap1 allow for regeneration of the inducer sensor.

## 2 Conclusions

There is no longer any scepticism regarding the existence in the cell of ‘ … *a protein endowed with highly reactive cysteine residue(s) that serves as the sensor for small-molecule inducers of the phase 2 response*’. Several decades of research by multiple independent groups have convincingly demonstrated that this protein is Keap1. Furthermore, the extraordinary ability of Keap1 to accommodate inducers of many different shapes and sizes, and with varying degrees of reactivity, has given rise to a ‘cysteine code’, which ensures fine tuning and tight control of the Keap1/Nrf2 stress-sensing response. This response is the intended target for small molecule activators of several different chemical classes, some of which are currently in clinical trials. Further research is essential for detailed understanding of the precise consequences of targeting Keap1 for disease prevention and treatment. Acknowledgments We are extremely grateful to Cancer Research UK (C20953/A18644) and the BBSRC (BB/J007498/1) for financial support.
